# *Neisseria lactamica* Y92–1009 complete genome sequence

**DOI:** 10.1186/s40793-017-0250-6

**Published:** 2017-07-24

**Authors:** Anish K. Pandey, David W. Cleary, Jay R. Laver, Martin C. J. Maiden, Xavier Didelot, Andrew Gorringe, Robert C. Read

**Affiliations:** 1 Faculty of Medicine, Southampton NIHR Biomedical Research Centre and Institute for Life Sciences, University of Southampton, Southampton, UK; 2 Department of Zoology, University of Oxford, South Parks Road, Oxford, UK; 3 Department of Infectious Disease Epidemiology, Imperial College London, London, United Kingdom; 4 Public Health England, Porton Down, UK

**Keywords:** SMRT cell sequencing, *Neisseria*, Short read sequencing, Bacteria, Genome assembly, Nasopharyngeal microflora, Commensal

## Abstract

We present the high quality, complete genome assembly of *Neisseria lactamica* Y92–1009 used to manufacture an outer membrane vesicle (OMV)-based vaccine, and a member of the *Neisseria* genus. The strain is available on request from the Public Health England Meningococcal Reference Unit. This Gram negative, dipplococcoid bacterium is an organism of worldwide clinical interest because human nasopharyngeal carriage is related inversely to the incidence of meningococcal disease, caused by *Neisseria meningitidis*. The organism sequenced was isolated during a school carriage survey in Northern Ireland in 1992 and has been the subject of a variety of laboratory and clinical studies. Four SMRT cells on a RSII machine by Pacific Biosystems were used to produce a complete, closed genome assembly. Sequence data were obtained for a total of 30,180,391 bases from 2621 reads and assembled using the HGAP algorithm. The assembly was corrected using short reads obtained from an Illumina HiSeq 2000instrument. This resulted in a 2,146,723 bp assembly with approximately 460 fold mean coverage depth and a GC ratio of 52.3%.

## Introduction

*Neisseria lactamica* (henceforth, *Nla)* is a Gram negative, diplococcoid, commensal organism that colonises the human nasopharynx. In common with other commensal *Neisseria* *spp.,* including *Neisseria mucosa* *,* *Neisseria. sicca* *and* *Neisseria cinerea* *,* carriage of *Nla* very rarely leads to invasive disease, and then only in severely immunocompromised individuals [1]. Examples of more commonly pathogenic *Neisseria* lineages include the causative agents of gonorrhoea and invasive meningococcal disease (IMD), *N. gonorrhoeae* (*Ngo)* and *N. meningitidis* *(Nme),* respectively [2]. *Nla* is biochemically differentiated from other members of the genus *Neisseria* by its ability to produce β-D-galactosidase and therefore ferment lactose.

*Neisseria lactamica* Y92–1009 was originally isolated during a school carriage study in Northern Ireland in 1992 and has been assigned sequence type 3493 and belongs to the ST-613 clonal complex. The strain has been used for various purposes over the past 15 years. For example, it has been used to manufacture an OMV based vaccine intended to protect against against *Nme* [3] and used in experimental human challenge studies [4, 5] where it was shown to inhibit co-colonisation carriage rates of the potentially pathogenic meningococcus. The general properties of the genus, species and strain are presented in Table 1.

**Table 1:** Classification and general features of *Neisseria lactamica* strain Y92–1009 according to MIGS specification [36]

MIGS ID	Property	Term	Evidence code^a^
	Classification	**Domain** Bacteria	TAS [37]
		**Phylum** *Proteobacteria*	TAS [37]
		**Class** *Betaproteobacteria*	TAS [37]
		**Order** *Neisseriales*	TAS [37]
		**Family** - *Neisseriaceae*	TAS [37]
		**Genus** *Neisseria*	TAS [37]
		**Species** lactamica	TAS [37]
		**Strain**: Y92–1009 (Accession)	
	Gram stain	Negative	IDA
	Cell shape	Diplococcus	IDA
	Motility	Non-motile but piliated	TAS [38]
	Sporulation	Not reported	NAS
	Temperature range	32–39 °C	IDA
	Optimum temperature	37.0 °C	IDA
	pH range; Optimum	3.5–6.5 °C; 5 °C	TAS
	Carbon source	Glucose, Maltose, Lactose	TAS [39]
MIGS-6	Habitat	Human Nasopharynx	TAS [37, 39]
MIGS-6.3	Salinity	0.9%	TAS [39]
MIGS-22	Oxygen requirement	Aerobe	TAS [39]
MIGS-15	Biotic relationship	commensal	TAS [39]
MIGS-14	Pathogenicity	Non-pathogen	TAS [39]
MIGS-4	Geographic location	Londonderry, Northern Ireland	TAS [40]^b^
MIGS-5	Sample collection	1992	TAS [40]^b^
MIGS-4.1	Latitude	54.9966 N	NAS
MIGS-4.2	Longitude	7.3086 W	NAS
MIGS-4.4	Altitude	128 m	NAS

^a^Evidence codes - IDA: Inferred from Direct Assay; TAS: Traceable Author Statement (i.e., a direct report exists in the literature); NAS: Non-traceable Author Statement (i.e., not directly observed for the living, isolated sample, but based on a generally accepted property for the species, or anecdotal evidence). These evidence codes are from the Gene Ontology project [2]

^b^Data for isolate geographic location and sample collection was acquired by searching for *N. latamica* Y92–1009 (ID number: 4945) on pubMLST *Neisseria*

A study of asymptomatic carriage of *Nme* and *Nla* in 2969 healthy infants and children demonstrated that carriage rates of *Nla* were highest in 18 month old infants and declined to a much lower rate in teenage children [6]. Conversely, a low level of meningococcal carriage was detected in infants during the first 4 years of life with increased carriage in those aged between 14 and 17. These findings have been confirmed by further studies including the Stonehouse survey [7], which reported that *Nla* carriage was six times higher in children up to the age of 5 years old, with relatively lower carriage rates for *Nme*. Similar results were also observed in a more recent study [8]. Taken together, these studies suggest a protective role for *Nla* against meningococcoal disease by preventing meningococcal colonization in younger children. Harnessing this natural carriage dynamic has been proposed as a potential strategy to reduce *Nme* carriage, a pre-requisite for invasive meningococcal disease [9].

## Organism information

### Classification and features

**Fig. 1:**
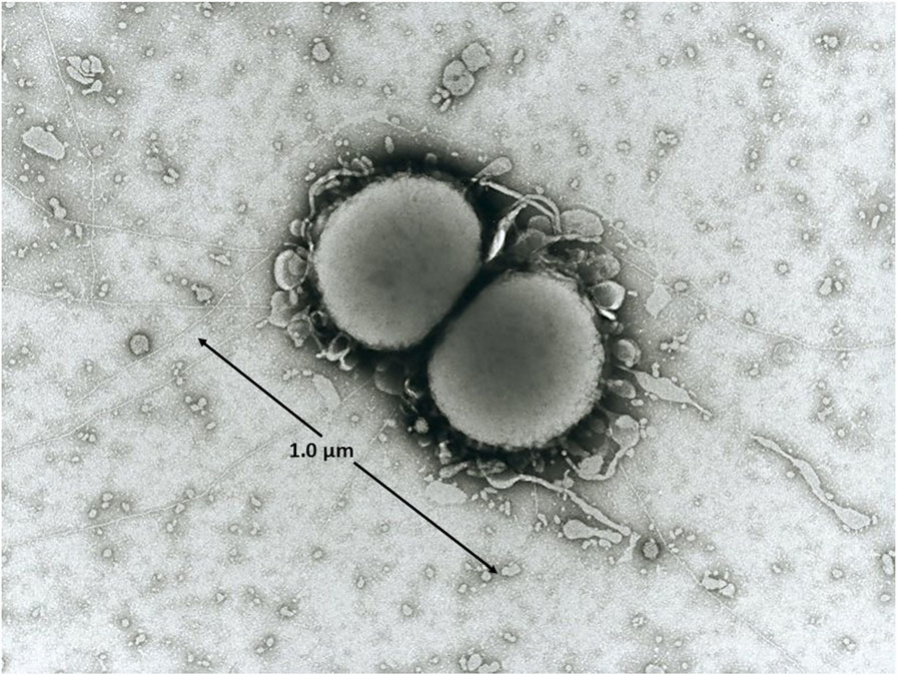
Photomicrograph of *N. lactamica* Y92–1009. This image was obtained with transmission electron micrography and displays the diplococcoid nature of the *N. lactamica* Y92–1009 cell. The size of the cell is indicated in micrometres (μm)

*Nla* is a Gram negative, non-sporulating, diplococcoid bacterium. Bacterial cells are approximately 1 μM in diameter. An electron micrograph, generated by staining with a 1% potassium phosphotungstate (PTA) for 5–10 s and captured using a Philips EM300 with an accelerating voltage of 60 kV, is shown in Fig. 1. On solid media the organism forms unpigmented to yellow, smooth and transparent colonies. The cells excrete outer membrane vesicles (OMVs) approximately 100 nm in diameter. In liquid culture, this species is likely to aggregate. Like other members of its genus*, Nla* is oxidase positive, catalase positive and successfully reduces nitrite (NO_2_^−^) ions. *Nla* can be differentiated from the other members of the genus by its ability to ferment lactose and produce β-D-galactosidase. *Nla, like Nme,* dwells within the human nasopharynx but is more commonly found in infants and young children. It has been isolated from the urogenital tract on one occasion [10] but is almost never associated with disease [1].

*Nla* unlike *Nme* is known to autoagglutinate and as such cannot be classified into serogroups. A multi locus sequence typing approach (MLST) uses the allelic profiles of seven, indexed housekeeping genes (*abcZ, adk, aroE, fumC, gdh, pdhC & pgm)* to classify a *Neisseria* isolate into a sequence types (ST) as reported in the database Pubmlst.org/neisseria [11].

At the time of writing a total of 21 unique ST’s of *Nla* have been described, which have been sub-classified into 6 clonal complexes (ccs). The cc640 includes ST’s 10,984, 10,326, 11,143 & the *Nla* 020–06 reference genome and the cc613 contains ST 11653 and *Nla* Y92–1009, the isolate described here. The cc595 contains ST 595, and cc624 contains ST’s 6206, 624, & 11,383. Finally, cc1494 contains ST’s 642, 1494 & 1495. To differentiate *Neisseria* strains further typing schemes have been developed using variable regions of hypervariable loci such as *porA* and *fetA* [12].

**Fig. 2:**
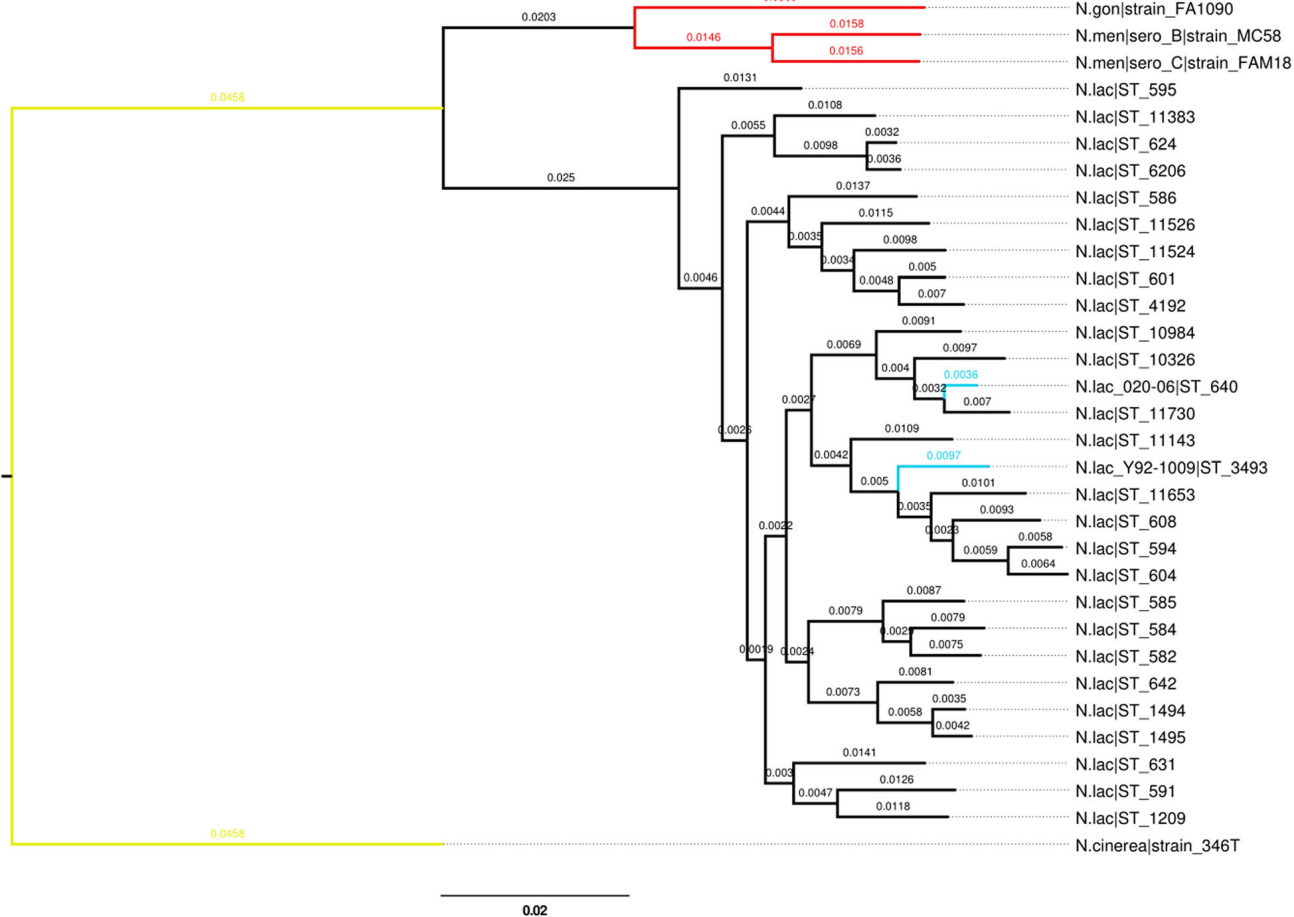
Phylogenetic tree indicating the position of *N. lactamica* Y92–1009 amongst other pathogenic and commensal *Neisseria*. This tree was constructed based on a core genome comparison of a collection of 32 *Neisseria* assemblies generated using the genome comparator tool available on pubmlst.org/neisseria. The reconstructed evolutionary relationships among *N. meningitidis* (*Red*, *n* = 2), N. gonnorhoeae (*Red*, *n* = 1), *N. cinerea* (*Yellow*, *n* = 1) and *N. lactamica* (*Black*, *n* = 27) are shown. The genome sequenced here, *N. lactamica* Y92–1009 is outlined in *cyan* (Nla_PacBio|ST_3493). This analysis included the sequenced genome and the best representative assembly for every identified sequence type (ST) of this species. The tree was generated using FastTree v.2 [13] and edited using Figtree v.1.4.3 [14]. The tree is drawn to scale, with branch length units being expressed as an overall proportion of divergence based on the comparison of 1629 genes

A core, gene by gene (*n* = 1629) MAAFT alignment of a collection of 32 whole genome *Neisseria* assemblies was done using the genome comparator tool available on Pubmlst.org/neisseria (Fig. 2). This included an example of the most complete representative assembly for every identified ST of *Nla* (*n* = 26) as well as the closed, reference genome *Nla* 020–06. These *Nla* isolates were compared to a cohort of pathogenic *Neisseria* reference genomes including *Nme* isolate MC58 (Serogroup B), *Nme* isolate FAM-18 (Serogroup C) and *Ngo* isolate FA1090. In addition to this the phylogenetic tree was rooted with another commensal *Neisseria* of interest*, Nci* 346 T. The comparison resolved the pathogenic *Neisseria* by their respective species and further sub-defined isolates belonging to the six clonal complexes of *Nla.* The comparison was calculated based on a core genome of 1629 genes. The maximum likelihood tree was generated using FastTree v.2.0 [13] (gtr nt model) and edited using Figtree v.1.4.3 [14].

## Genome sequencing information

### Genome project history

*Nla* Y92–1009 GMP stocks were generated in 2011 [4]. Chromosomal DNA was extracted at the University of Southampton and sent to TGAC, Norwich for SMRT cell sequencing (PacBio RS II) and the Wellcome Trust Genomics Centre, Oxford for short read sequencing using HiSeq 2000. The genome was assembled using the internal pipeline at TGAC. The assembly was analysed, error-corrected, annotated and utilised for downstream applications by the authors. The assembly process and genome statistics are summarized in Tables 2 and 3.

**Table 2:** Project information

MIGS ID	Property	Term
MIGS 31	Finishing quality	Complete
MIGS-28	Libraries used	SMRTbell Template prep kit
MIGS 29	Sequencing platforms	Pacific Biosciences RSI
MIGS 31.2	Fold coverage	470×
MIGS 30	Assemblers	HGAP
MIGS 32	Gene calling method	Prokka, Blast2GO
	Locus Tag	
	Genbank ID	CP019894
	GenBank Date of Release	17/02/2017
	GOLD ID	-
	BIOPROJECT	PRJNA331097
MIGS 13	Source Material Identifier	-
	Project relevance	Medical, Biotechnological

**Table 3:** Genome statistics

Attribute	Value	% of Total
Genome size (bp)	2,146,723	100
DNA coding (bp)	1,831,541	85.3
DNA G + C (bp)	1,123,594	52.3
DNA scaffolds	1	100
Total genes	2053	100
Protein coding genes	1980	96.4
RNA genes	72	3.5
Pseudo genes	16	0.8
Genes in internal clusters	16	0.8
Genes with function prediction	1918	93.4
Genes assigned to COGs	1527	74.3
Genes with Pfam domains	5	0.2
Genes with signal peptides	0	0
Genes with transmembrane helices	0	0
CRISPR repeats	3	0.1

The accession numbers associated with this genome are Bioproject (PRJNA331097) and Biosample (SAMN05437355) and Genbank (SUB1713102: pending assignation of genbank ID).

### Growth conditions and genomic DNA preparation

Frozen *Nla* Y92–1009 stock was plated onto Columbia agar supplemented with horse blood and grown overnight at 37 °C, 5% CO_2_. β-galactosidase activity, and therefore identity as *Nla*, was confirmed by exposure of colonies to 5-bromo-4chloro-3-indolyl-β-D-galactopyranoside (X-Gal) in phosphate buffered saline. Blue colonies were sub-cultured into Trypticase soy broth +0.2% yeast extract. The cultures were grown overnight at 37 °C, 5% CO_2_ at 320 rpm. The Gentra Puregene yeast/bacteria kit and protocol (Qiagen, UK) was used per manufacturer’s instructions to extract high molecular weight (>40 kb) DNA, assessed via gel electrophoresis and improving sample quality for long read sequencing.

DNA purity was initially assessed using a nanodrop 1000 spectrophotometer and then quantified using the Qubit 2.0 fluorometer and BR dsDNA kit (Invitrogen, UK). The resulting DNA samples were stored at four assessed by examining both the trace and absorbance levels of the 260/280 and 260/230 absorbance ratios in a nanodrop 1000 spectrophotometer. The resulting DNA samples were placed in −20 °C cryostorage.

Purified DNA samples were collected until a threshold of 30 μg DNA was reached. The sample (260/280: 1.83, 260/230: 1.86) was collated and sent to TGAC, Norwich for de novo long read sequencing.

### Genome sequencing and assembly

Following sample collection, TGAC reassessed the sample purity and performed the long read sequencing using the PacBio RSII instrument. Four SMRT cells, each sequencing 50,000 × 8500 bp length reads, were used. The HGAP assembler [15] was used to generate a closed genome sequence of 2191181 bases.

Illumina paired-end, short read (151 bp) HiSeq 2000 sequencing was carried out using the same stock as that sent for Pacific Biosciences RS II long read sequencing. Sequencing libraries for paired end sequencing were constructed using the EBNext DNA sample Prep Master Mix Set 1 Kit (New England Biolabs).

Following generation of sequencing reads, nextera adapter sequence was trimmed using trimmomatic V.0.36 [16]. These reads were mapped to the unedited HGAP assembly and used to detect and correct errors present in repetitive regions using Pilon v.1.17 [17]. The reads were also used to trim low coverage areas present at the beginning and end of the circular genome sequence using the Breseq pipeline v.0.26a [18]. This reduced the assembly size down to 2,146,723 bp. Assembly metrics were evaluated using Quast [19].

### Genome annotation

The Prokka pipeline [20] was used to putatively assign genetic function and identify RNA and pseudogenes. As part of this annotation pipeline, prodigal [21] was initially used to identify all co-ordinates of CDSs from the assembly but did not assign a putative gene product. Once all CDSs were detected, gene prediction is normally inferred by comparing an unknown protein to a database containing known protein sequences. To ensure maximum possible accuracy, this sequence-database homology comparison is staggered hierarchically in the following way by Prokka.

All putative CDSs are matched with a trusted list of proteins. From the only manually curated *Nlatamica* reference genome [22]. All unannotated proteins are then compared to the uniprot bacterial database, a *Neisseria* specific RefSeq database (enabled with the –genus and –usegenus flags) Interproscan searches were conducted using BLAST2GO software and searched against the MMMPFAM [23] SignalPHMM [24] and THHMM [25] databases to identify genes with PFAM domains, signal peptides and transmembrane helices respectively.

Any genes in internal clusters were identified using CD-HIT [26] using file of in silico translated proteins from identified ORFS. Any CRISPRs were found using CRISPRfinder [27].

## Genome properties

The *Nla* Y92–1009 genome assembly contains 2,146,723 bp with approximately 460 fold mean coverage depth and 52.3% GC ratio. The assembly was predicted to contain 2053 putative ORFs, 1980 of which code for proteins. There were 72 genes predicted to encode RNA genes and three CRISPR repeats were detected.

**Fig. 3:**
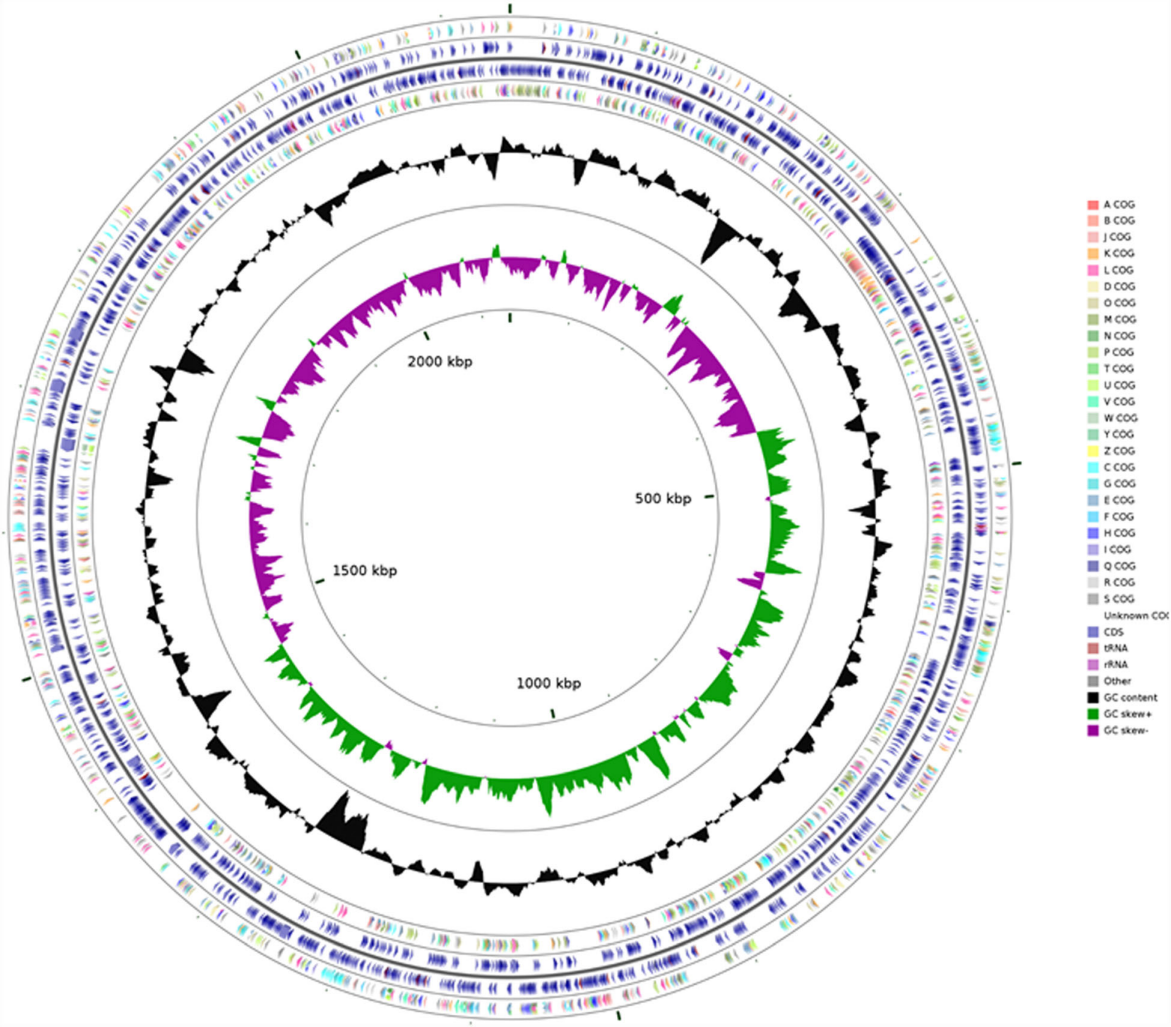
Circular map of *N. lactamica* Y92–1009 genome and features generated with Cgview Comparison Tool. The *arrows* in the outermost ring indicate putative genes (with the arrow indicating the 5′ to 3′ direction on the positive strand.) identified and assigned to Clusters of Orthologous Groups (COGs). The 20 COG categories are indicated by different colours from *Red* to *Grey* according to the colour key. The *second* and *third rings* indicate regions containing coding sequence (*Blue*), tRNA (Orange), and other RNAs (*Grey*), with the second ring running 5′ to 3′ (positive strand) and the third ring running 3′to 5′ (negative strand). The *fourth ring* indicates open reading frames assigned as COGs and encoded on the negative strand. The *fifth, black, graph ring* displays GC content while the *last ring* (*purple* and *green*) shows positive (*Green*) and negative (*Purple*) GC skew

Furthermore, 74.3% of total putative ORFs matched with the COG database; these results are presented in Table 4 and displayed in a circular genome diagram in Fig. 3.

**Table 4:** Number of predicted genes associated with general COG functional categories

Code	Value	%age	Description
J	148	7.21	Translation, ribosomal structure and biogenesis
A	1	0.05	RNA processing and modification
K	56	2.73	Transcription
L	137	6.67	Replication, recombination and repair
B	1	0.05	Chromatin structure and dynamics
D	24	1.17	Cell cycle control, Cell division, chromosome partitioning
V	23	1.12	Defense mechanisms
T	25	1.22	Signal transduction mechanisms
M	130	6.33	Cell wall/membrane biogenesis
N	20	0.97	Cell motility
U	42	2.05	Intracellular trafficking and secretion
O	75	3.65	Posttranslational modification, protein turnover, chaperones
C	109	5.31	Energy production and conversion
G	48	2.34	Carbohydrate transport and metabolism
E	129	6.28	Amino acid transport and metabolism
F	45	2.19	Nucleotide transport and metabolism
H	76	3.70	Coenzyme transport and metabolism
I	51	2.48	Lipid transport and metabolism
P	77	3.75	Inorganic ion transport and metabolism
Q	10	0.49	Secondary metabolites biosynthesis, transport and catabolism
R	137	6.67	General function prediction only
S	163	7.94	Function unknown
-	526	25.62	Not in COGs

The total is based on the total number of protein coding genes (1980) putatively discovered in the genome

## Insights from the genome sequence

This genome sequence was typical for a *Neisseria* genome, being small (2.2 Mb) with a high amount of coding content (85.3%).

Nla remains relatively poorly defined at the genomic level, with the COG analysis (Table 4) demonstrating that approximately a third (33.5%) of all open reading frames (ORFs) in this genome have either unknown, predicted function or did not possess sufficient homology to be sorted into a COG category. Additionally, there were only five matches when comparing *Nla* Y92–1009 translated ORFs against the PFAM database as well as a total lack of matches with SignalP and THHMM databases. Despite this, five ORFs presenting were annotated as putative signal peptides by Prokka.

Repetitive sequences play important roles in *Neisseria* genome modification and gene expression. The ten base pair DNA uptake sequence (DUS) has been shown to be pre-requisite for transformation in *Neisseria* species [28]. DUS-containing sequences have permeated the *Neisseria* genus core genome, indicating these sequences can survive genome diversification via recombination [29]. Another repeat type is named dRS3. This is an abundantly recurring 20 bp repeat sequence known to flank larger repeat sequences and act as a site for phage integration [30]. Finally, the transposon-like Correia repeat enclosed elements (CREEs) may combine with native sequence to form gene promotors as well as affect post transcriptional gene expression [31]. Therefore these elements have often been observed as hot spots of DNA rearrangement and recombination [32]. Repeat sequence content also reflects on the evolutionary history of an organism. *Nme* possesses many more CREEs than any other member of the genus. This is thought to have arisen after the species diversified away from a common ancestor [33].

Motifs for these repetitive sequences were taken from a genus wide study of *Neisseria* that reported DUS, CRE and dRS3 motifs among ten species [34] and a study on the overepresentation of DUS motifs (dialects)among the *Neisseriaceae* [28]. These motifs were searched in the genome using fuzznuc as part of the EMBOSS [35] package. The frequency of these repetitive sequence motifs observed in *Nla* Y92–1009 is described in Table 5. In comparison to isolates examined in the first study cited [34], *Nla* isolate Y92–1009 has a higher number (*n* = 454) of dSR3 elements than all other neisserial species bar *Nme* isolate MC58 (*n* = 689). This value also exceeded the number those detected in another *Nla* isolate ATCC 23970 (*n* = 197) and *N. gonnorhoeae* isolate FA 1090 (*n* = 208). *Nla* isolate Y92–1009 exhibited a lower numbers of CREE repeats (*n* = 86) than all other known *Neisseria* except for *Nme* isolate MC58 (*n* = 524) (Table 5).

**Table 5:** Frequency of repeat sequences in *N. lactamica* Y92–1009 genome

Repeat type	Repeat sequence	Value
AT-DUS	‘ATGCCGTCTGAA’	1718
AG-DUS	‘AGGCCGTCTGAA’	262
AG-mucDUS	‘AGGTCGTCTGAA’	45
DSR3	‘ATTCCCNNNNNNNNGGGAAT’	454
Correia	‘ATAG[CT]GGATTAACAAAAATCAGGAC’	50
	‘TATAG[CT]GGATTAAATTTAAACCGGTAC’	1
	‘TATAG[CT]GGATTAACAAAAACCGGTAC’	17
	‘TATAG[CT]GGATTAAATTTAAATCAGGAC’	17

In comparison to isolates examined in the *Neisseria* *-*wide DNA uptake sequence study, *Nla* isolate Y92–1009 possessed an overrepresentation of AT-DUS sequence (*n* = 1718) which was typical of other *Nla* isolates and the pathogenic *Neisseria* *.* It also possessed low levels of AG-DUS and AG-mucDUS dialects which are found more prominently in species such as *N. polysaccherea*, *N. cinerea* *(Nci)* and *N. mucosa* *.* Due to the interspecific barrier to transformation that exists between *Neisseria* spp. with uncomplimentary DUS dialects, it is more likely that *Nla* would engage in transformation with *Neisseria* *spp.* possessing the same DUS dialect.

**Fig. 4:**
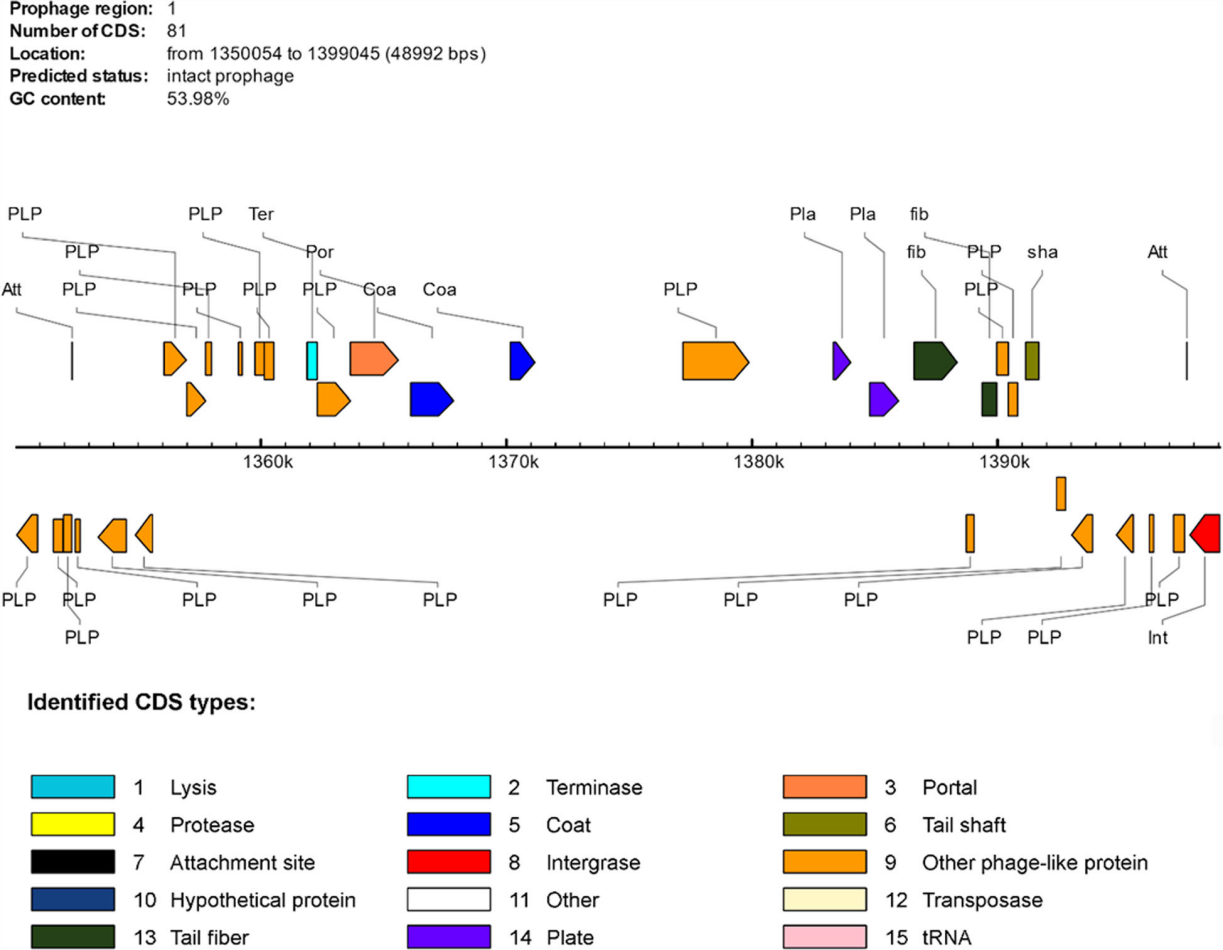
Graphic showing phage related proteins identified in the intact prophage by PHAST. The diagram above was annotated by and imported from the PHAST-prophage database. Twenty-six hypothetical proteins were removed from the schematic to increase the clarity of the phage related proteins. The proteins above the *black line* indicating genome position are encoded 5′ to 3′ while the proteins under it are encoded 3′ to 5′. The abbreviations in the diagrams are as follows **Att** (phage attachment site), **Coa** (Phage coat protein), **fib** (Phage Tail Fiber), **Int** (Phage integrase) **Pla** (Phage plate protein), **PLP** (Phage like protein), **Por** (Portal protein) **sha** (Phage tail shaft protein) **Ter** (Terminase)

A number of phage related genes and atypical GC content were seen in a region starting 1, 350, 054 bp and running until 1,399,045 bp. The presence of a prophage was investigated by PHAST (Phage Search Tool; http://phast.wishartlab.com/index.html). PHAST detected an intact prophage sequence 49.8Kb in length. This contained 53.98% GC content and possessed 81 proteins, 46 of which were phage associated. These proteins include two attachment sites, two coat proteins, two tail-fiber proteins, an integrase, two plate, a portal, a tail shaft and terminase subunits (Fig. 4 **)**. The prophage sequence scored a completeness score of 130, where 150 is the maximum and a minimum score of 90 indicates intactness of prophage. The proteins from the putative, *Nla* Y92–1009 prophage sequence were compared against the PHAST-prophage database. This revealed that at least one predicted protein from this prophage sequence was detected in over 23 different species of bacteriophage. Three of the four bacteriophages with the greatest number of shared proteins with the *Nla* Y92–1009 prophage were found in *Acinetobacter* phages. While these prophages only shared 18, 17 and 16 proteins respectively out of a potential 81, this stretch of sequence was found to be highly conserved.

## Conclusions

This closed whole genome assembly is the first for this specific strain of *Nla* isolate Y92–1009 and the second for the species as a whole and contains 2,146,723 bp, that encode 1980 predicted proteins, 72 RNA genes and three CRISPR repeats. The profile of repeat sequence patterns discovered in this genome compared to other *Neisseria* *spp.* indicates that it possesses DUS, CREE motifs and numbers typical to other *Nla* isolates but contains an unexpectedly high amount of dRS3 repeats for a commensal *Neisseria* *,* similar to the number seen in *Nme* isolate MC58. This genome will form the reference for studies on the microevolution of commensal *Neisseria* among individuals experimentally challenged with this strain.

## Abbreviations

Bp: Base pairs
Cc: Clonal complex
CDS(s): CoDing sequence
COG: Clusters of Orthologous Groups
CREEs: Correia repeat enclosed elements
CRISPR: Clusters of regularly intersperced short palindromic repeats
DUS: DNA uptake sequence
GC: Guanine-cytosine ratio
GMP: Good manufacturing practice
MLST: Multi locus sequence typing
*Nci*: *Neisseria cinerea*
*Ngo*: *Neisseria gonorrhoeae*
*Nla*: *Neisseria lactamica*
*Nme*: *Neisseria meningitidis*
ORFs: Open reading frames
PacBio: Pacific biosciences
SMRT: Smart cell sequencing
ST: Sequence type
V.(#): Program Version followed by number
