# Correction to: *Neisseria lactamica* Y92–1009 complete genome sequence

**DOI:** 10.1186/s40793-017-0288-5

**Published:** 2018-02-16

**Authors:** Anish K. Pandey, David W. Cleary, Jay R. Laver, Martin C. J. Maiden, Xavier Didelot, Andrew Gorringe, Robert C. Read

**Affiliations:** 1 Faculty of Medicine, Southampton NIHR Biomedical Research Centre and Institute for Life Sciences, University of Southampton, Southampton, UK; 2 Department of Zoology, University of Oxford, South Parks Road, Oxford, UK; 3 Department of Infectious Disease Epidemiology, Imperial College London, London, UK; 4 Public Health England, Porton Down, UK

## Correction

After publication of the original article [1] it was identified that an incorrect version of the manuscript has been published. This was caused by a technical error which led to a discrepancy between the editorially-accepted version of the manuscript, and the published version.

A list of Corrections is included below:

1. The following abbreviations have been corrected throughout the article:
  *Nla* changed to *N. lactamica*
  *Nme* changed to *N. meningitides*
  *N.go* changed to *N. gonorrhoeae*
  *Nci* changed to *N. cinerea*
2. Table 1: ‘Term’ and ‘Evidence code’ columns of the first 7 rows have been updated to the following:

**Table:** 

Domain Bacteria	TAS [37]
Phylum *Proteobacteria*	TAS [38]
Class *Betaproteobacteria*	TAS [39, 40]
Order *Neisseriales*	TAS [39, 41]
Family - *Neisseriaceae*	TAS [42]
Genus *Neisseria*	TAS [42]
Species *Neisseria lactamica*	TAS [42, 43]

3) Table 2: Missing/blank term for MIGS 13 has been changed to read “Strain is not publicly available”.
4) 2nd paragraph under heading “Insights from the genome sequence”: Sentence containing “…approximately a third (33.5%) of all open reading frames (ORFs) in this genome…” has been shortened to “approximately a third (33.5%) of all ORFs in this genome…”
5) 3rd paragraph under heading “Insights from the genome sequence”: Bracketed abbreviation ‘(DUS)’ has been removed.
6) 3rd paragraph under heading “Insights from the genome sequence”: Bracketed abbreviation ‘(CREEs)’ has been removed
7) Bracketed link for PHAST ‘(http://phast.wishartlad.com/index.html)’ changed to reference 35.
8) Reference 14 initially missing from article, has now been added and correctly referenced
9) Reference 33 was removed, as it was a duplicate of reference 30.
10) References 36–40 removed, and replaced with updated reference list (36–46):

36. Field D, Garrity G, Gray T, Morrison N, Selengut J, Sterk P, et al. The minimum information about a genome sequence (MIGS) specification. Nat Biotechnol. 2008;26:541–7.

37. Woese CR, Kandler O, Wheelis ML. Towards a natural system of organisms: proposal for the domains *Archaea*, *Bacteria*, and *Eucarya*. Proc Natl Acad Sci. 1990;87:4576–9. 10.1073/pnas.87.12.4576.

38. Garrity GM, Holt JG. The Road Map to the Manual. In: Bergey’s Manual® of Systematic Bacteriology. 2001. p. 119–66. 10.1007/978-0-387-21609-6_15.

39. Euzeby. J. List of new names and new combinations previously effectively, but not validly, published. Int J Syst Evol Microbiol. 2006;56:1–6.

40. Garrity GM, Bell JA, Lilburn T. Class II. *Betaproteobacteria* class. Nov. In: Bergey’s Manual® of Systematic Bacteriology. 2005. p. 575–922. 10.1007/0-387-29298-5_2.

41. Tønjum T. *Neisseriales* ord. Nov. In: Bergey’s Manual of Systematics of Archaea and Bacteria. John Wiley & Sons, Ltd.; 2015. 10.1002/9781118960608.obm00079.

42. SKERMAN VBD, McGOWAN V, SNEATH PHA. Approved Lists of Bacterial Names. Int J Syst Evol Microbiol. 1980;30:225–420.

43. Hollis DG, Wiggins GL, Weaver RE. *Neisseria lactamicus* sp. n., a lactose-fermenting species resembling Neisseria meningitidis. Appl Microbiol. 1969;17:71–7.

44. Aho EL, Keating a M, McGillivray SM. A comparative analysis of pilin genes from pathogenic and nonpathogenic Neisseria species. Microb Pathog. 2000;28:81–8.

45. Lennette, Edwin H. Manual of Clinical Microbiology. 4th edition. Washington DC: American Society for Microbiology; 1985.

46. Neisseria MLST website. https://pubmlst.org/neisseria/. Accessed 2 Feb 2017.

The Publisher apologises for this error.
